# Transitioning from antenatal surveillance surveys to routine HIV testing: a turning point in the mother-to-child transmission prevention programme for HIV surveillance in Brazil

**DOI:** 10.1186/s12879-017-2540-4

**Published:** 2017-07-05

**Authors:** Gerson Fernando Mendes Pereira, Meritxell Sabidó, Alessandro Caruso, Adele Schwartz Benzaken

**Affiliations:** 1 Department of STI, AIDS and Viral Hepatitis, Secretary for Health Surveillance, Ministry of Health Brazil, SAF Sul, Trecho 02, Bloco F, Torre 1, Edifício Premium, Auditório, Brasília DF, CIE CEP 70070-600 Brazil; 2 TransLab. Department of Medical Sciences, Universitat de Girona, Catalonia, Spain; 3 CIBER Epidemiology and Public Health (CIBERESP), Madrid, Spain

**Keywords:** HIV, Pregnant women, Surveillance, Brazil

## Abstract

**Background:**

In Brazil, due to the rapid increase in programmes for the prevention of mother-to-child transmission (PMTCT), routine programme data are widely available. The objective of this study was to assess the utility of programmatic data to replace HIV surveillance based on the antenatal care (ANC) surveillance survey (SS).

**Methods:**

We analysed ANC SS data from 219 maternity service clinics. PMTCT variables were extracted from the ANC SS data collection form, which allowed us to capture and compare the ANC SS data and PMTCT HIV test results for each pregnant woman who completed the ANC SS. Both the PMTCT programme and the ANC SS tested for HIV using sequential ELISA and western blot for confirmation. We assessed the completeness (% missing) of the PMTC data included in the ANC SS.

**Results:**

Of the 36,713 pregnant women who had ANC SS HIV tests performed, 30,588 also underwent PMTCT HIV testing. The HIV prevalence rate from routine PMTCT testing was 0.36%, compared to 0.38% from the ANC SS testing (relative difference −0.05%; absolute difference −0.02%). The relative difference in prevalence rates between pregnant women in northern Brazil and pregnant women central-west Brazil was −0.98 and 0.66, respectively. Of the 29,856 women who had HIV test results from both the PMTCT and ANC SS, the positive percent agreement of the PMTCT versus the surveillance test was 84.1% (95% confidence interval [CI]: 74.8–91.0), and the negative percent agreement was 99.9% (95% CI: 99.9–100.0). The PMTCT HIV testing uptake was 86.4%. The ANC SS HIV prevalence was 0.33% among PMTCT non-refusers and 0.59% among refusers, with a percent bias of −10.80% and a differential prevalence ratio of 0.56. Syphilis and HIV testing results were complete in 98% and 97.6% of PMTCT reports, respectively. The reported HIV status for the women at clinic entry was missing.

**Conclusions:**

Although there were consistent HIV prevalence estimates from the PMTCT data and the ANC SS, the overall positive percent agreement of 84.1% falls below the World Health Organization benchmark of 94.7%. Therefore, Brazil must continue to reinforce data collection practices and ensure the quality of recently introduced rapid HIV testing before replacing the PMTCT data with surveillance techniques. However, some regions with better results could be prioritized to pilot the use of PMTCT data for surveillance.

## Background

Brazil has a stable HIV/AIDS epidemic at the country level that is concentrated in key populations [1]. The adult prevalence is estimated to be 0.38% at the national level [2], although it displays distinct regional HIV burdens and trends [1].

The Brazilian government has conducted periodic and anonymous probability sample surveys to determine the HIV prevalence among parturient women using public sector maternity services since 1996. [2–8]. Data from these surveys are used to monitor HIV trends in pregnancy and are applied as a proxy to estimate the prevalence of HIV among adults in the general population [9]. Moreover, these data are used to make projections about HIV incidence that are helpful to allocate resources and to evaluate the effectiveness of the surveillance system for identifying HIV cases among this population [10, 11].

In Brazil, each antenatal (ANC) HIV surveillance survey (SS) tested parturient women tested for HIV [3, 5–7], except in 2006, during which time the HIV prevalence was calculated from routinely collected data at facilities at the local level [4]. PMTCT variables were collected through a specific form, and the results of the ANC SS indicate that the HIV national prevalence rate has remained stable, at approximately 0.4%, although the testing of pregnant women has increased from 52% [3] to 86.6% [2].

In Brazil, the PMTCT programme is integrated into the ANC services. The PMTCT national protocol recommends the testing of all pregnant women using sequential points-of-care (POC) for syphilis and HIV testing and counselling during the first ANC visit or during the first trimester [12]. Pregnant women whose HIV tests are non-reactive are considered HIV-negative, but they are tested again during the third trimester. When a positive HIV result is confirmed, women are initiated on antiretroviral drugs after being referred from the ANC programme to the ART care settings [12]. Before the implementation of POC testing as part of the ANC services in 2012 [13], the PMTCT HIV diagnostic algorithm used fourth-generation ELISA data confirmed by western blotting [14]. ANC coverage in Brazil is high (98.5%); therefore, women who attend ANC are considered representative of all pregnant women in the country [2].

As a result of an increase in PMTCT strategies, Brazil has considered transitioning to the use of routinely collected HIV testing data to estimate the HIV prevalence among pregnant women. High-quality routinely collected PMTCT programme data could supplement or replace traditional ANC SS at a significant lower operational and monetary cost.

The World Health Organization (WHO) recommends assessing five core elements of PMTCT programme data and quality before a successful transition from ANC SS to surveillance using routine antenatal HIV testing can be considered [15]. These elements are as follows: (1) agreement between ANC SS and routine antenatal HIV test results; (2) magnitude of selection bias inherent in PMTCT-based HIV estimates; (3) the coverage of PMTCT routine HIV testing services at ANC SS sites; (4) the quality of a minimum set of surveillance variables in routinely collected programme records; and (5) quality assurance practices of HIV testing at ANC sites.

To assess Brazil’s readiness to use routinely collected antenatal HIV testing data for ANC based surveillance, we conducted a cross-sectional evaluation in all ANC sites participating in the 2010–2012 round of ANC SS.

## Methods

### Study design

The analysis of existing data collected through the ANC SS to assess the utility of PMTCT programme data for surveillance among pregnant women had two components.

The first component consisted of a comparison of ANC SS HIV test results and prevalence estimates with those obtained from routine PMTCT HIV testing during the same period. With respect to the PMTCT, the first HIV test conducted during the pregnancy was considered, and the PMTCT variables were extracted from the ANC SS data collection form. This method allowed us to capture and compare ANC SS and PMTCT HIV test results for each pregnant woman sampled by ANC SS to quantify PMTCT HIV testing uptake and explore potential bias in PMTCT-based HIV prevalence estimates.

The second component was consistent with the assessment of the completeness of routine PMTCT data collected during the ANC SS period as determined based on the proportion of missing data.

### Study sites and study population

The study sites included in this assessment included 219 public maternity services across the country that participated in the ANC SS between October 2010 and 2012.

The study population consisted of all women who participated in the ANC SS. This survey prospectively enrolled pregnant women admitted for delivery who were between 15 and 49 years of age and had signed a written consent form to participate. From this study population, the current assessment included parturient women who had tested for HIV in the ANC SS. In the component aimed to compare HIV testing between the ANC SS and PMTCT programme data, data from women with both test results were analysed. Women with no PMTCT were excluded from the analysis. Furthermore, with respect to the PMTCT data quality assessment component, we analysed data from all participating parturient women who had been HIV tested as part of the ANC SS.

### Sampling strategy of the ANC HIV SS

The ANC HIV SS consisted of an estimated probability sample of 40,000 parturient women (8000 in each region) recruited at maternity public services from the Unified Health System (Sistema Único de Saúde, SUS) in Brazil. Maternity service wards offer health services for women in labour or delivery. A stratified two-stage cluster sampling design was used. In the initial stage of sample selection, a random sample of 219 public hospital maternity service wards with at least 500 deliveries in 2007 were considered the primary sampling unit. Maternity services were stratified according to each region (north, northeast, southeast, south, and central-west) and the population size of the municipality (< 50,000; 50,001 to 400,000; and ≥ 400,001 inhabitants) where each service was located. In each stratum, 15 public hospital maternity service wards were selected by a probability proportional to the number of admissions for delivery in 2007. In the second stage, within each maternity ward, a sample of 180 to 200 pregnant women were randomly selected upon admission for delivery.

### HIV ANC SS testing

For each parturient woman who consented to participate in the ANC survey, health workers collected eight drops of blood from a finger prick using S&S 903 sample collection cards (Schleicher & Schuell BioScience, Keene, NH, USA). The dried blood spot (DBS) samples were sent by mail in Zip-lock plastic bags with desiccant silica to the HIV/AIDS Research Laboratory of the Caxias do Sul University and stored at room temperature. The DBS samples were tested using an enzyme-linked immunosorbent assay (ELISA) (Q-Preven HIV 1+2 DBS, Symbiosis Diagnóstica, Leme, Brazil). All positive results were confirmed with a second ELISA and Western blot analysis (DAVIH-BLOT, Laboratorios DAVIH, La Habana, Cuba). Though the test results were not returned to the participants, they could retrieve their results online using a specific study number. Those who tested positive were referred to the nearest health care centre for counselling and for appropriate follow-up care.

### Extraction of PMTCT variables using the ANC SS data collection form

Sociodemographic and PMTCT variables were extracted from the ANC SS data collection form. During the ANC SS, trained health care workers completed an SS data collection form by abstracting information from the mother’s ANC card and register and by interviewing the participants using a structured questionnaire. Data were automatically recorded using the TeleForm 10.2 software (Cardiff Software Inc., Solana Beach, CA, USA). The extracted data included the following: (1) sociodemographic variables (age, municipality of residence, educational level, and self-declared race/colour); (2) the date and number of ANC visits; and (3) PMTCT-related information about the first and second HIV and VDRL tests (performance status, date, and result).

### Data analyses

Data were described using frequencies and results stratified by region. We compared estimates of the proportion of tests that were positive among pregnant women obtained from routine PMTCT data and ANC SS. For routine PMTCT data, the first HIV test performed during pregnancy was used. We calculated 95% confidence intervals (CIs) of the estimates of the proportion of tests that were HIV-positive using the exact method and adjusted standard errors for the potential of clustering on the ANC facility.

The individual-level agreement of PMTCT and ANC SS HIV testing results was calculated among all women with both test results. The positive percent agreement (PPA) and negative percent agreement (NPA) between test results were calculated using the ANC SS test as the reference. We measured the difference in prevalence between the PMTCT HIV status (R) and ANC SS (S). The absolute difference was determined as (R-S), whereas the relative difference was calculated as (R-S)/S.

We assessed the effect of selection bias on the HIV prevalence estimates from routine HIV testing using the percent bias or the percent change (positive or negative) from the total HIV prevalence measured in ANC SS with regard to the observed HIV prevalence in routinely tested women. This was calculated as the difference between the HIV prevalence among women sampled in the ANC SS who consented to routine HIV testing and the overall ANC SS prevalence divided by the overall ANC SS prevalence. We repeated the analysis for those with missing results in the PMTCT test and for those women with no missing results in PMTCT tests.

Two additional statistics were conducted to explore the magnitude and direction of selection bias. The first was the uptake of PMTCT HIV testing, defined as the proportion of pregnant women sampled by ANC SS who received PMTCT HIV testing defined as their first test during pregnancy. The second was the differential prevalence ratio defined as the ratio of HIV prevalence in women sampled in the ANC SS who received PMTCT HIV testing to the HIV prevalence for women sampled in the ANC SS who did not receive PMTCT HIV testing.

Because study participants were present in both the PMTCT and ANC SS groups, we considered them dependent samples. Therefore, statistical tests for the comparison of ANC SS based results and PMTCT based results could not be conducted; thus, only descriptive statistic results are presented. The completeness of various data elements included in routine PMTCT registers was estimated through the proportion of missing values.

All analyses were conducted with the complex survey functions of Stata 11 (StataCorp LP, College Station, TX, USA) and incorporated weighting, clustering (because clinics with different sizes were included), and stratification of data.

## Results

### Demographic characteristics of study sample

**Fig. 1:**
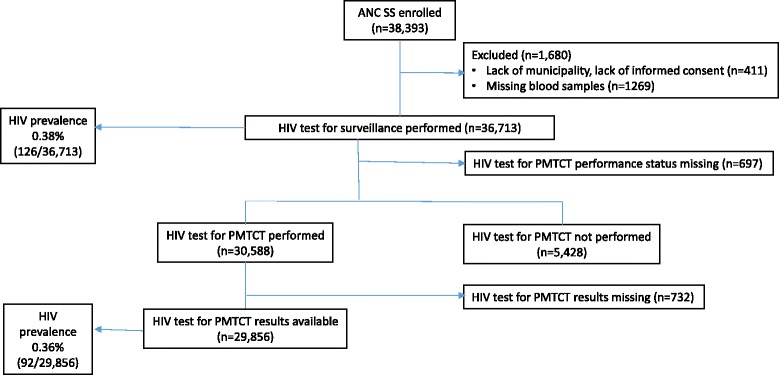
Antenatal care participants in prevention of mother-to-child transmission and antenatal care surveillance survey data. ANC: antenatal care; PMTCT: prevention of mother-to-child transmission; SS: surveillance survey

Figure 1 presents the study flow chart. A total of 38,393 (96%) parturient women were enrolled in the ANC SS. Of these, 1680 were excluded (4.4%) due to the lack of municipality information, which was important for calibration of the sample, the absence of a signed informed consent form, or missing blood samples. Thus, 36,713 pregnant women receiving maternity services across Brazil were considered for the analysis of ANC SS data (Fig. 1).

With respect to the analysis of the PMTCT data, 692 women who did not have information on whether a PMTCT was performed were excluded, resulting in the inclusion of 30,588 women. Furthermore, regarding the comparison of ANC SS and PMTCT data, we excluded 732 women who did not have PMTCT test results. Accordingly, 29,856 women had PMTCT and ANC SS test results available and were included in the comparison analysis.

Among women who underwent both HIV tests, 21.6% of were in the 15–19-year-old age group, and of these, 5.4% had not completed primary school, 24.5% lived in the southeast region, and 48.9% self-reported being of mixed race.

### Agreement between ANC SS and routine PMTCT HIV test results

In total, 0.36% (95% CI: 0.27–0.47) of women had an HIV-positive result from routine PMTCT HIV testing and 0.38% (95% CI: 0.31–0.48) had a positive result from ANC SS data (Table 1). In absolute terms, HIV prevalence was 0.02 percentage points lower when measured via routine PMTCT HIV testing than that when ANC SS HIV tests were performed on the same participants, representing a 0.05 relative decrease in the prevalence rate (Table 1). According to demographical variables, almost all categories showed similar prevalence estimates with overlapping CIs. By region, the northern and central-western areas presented the greatest differences in prevalence rates estimates between both tests with a relative difference in prevalence of −0.98 and of 0.66, respectively.

**Table 1:** HIV prevalence estimates from PMTCT data and ANC SS sites by demographic characteristics, Brazil 2010–2012

	PMTCT data			ANC SS data				
	Unweighted		Weighted	Unweighted		Weighted	Difference in prevalence	
	HIV +	Total	HIV prevalence (95% CI)	HIV +	Total	HIV prevalence (95% CI)	Relative	Absolute
Overall	92	29,856	0.36 (0.27–0.47)	126	36,713	0.38 (0.31–0.48)	−0.05	−0.02
Age in years								
15–19	11	6443	0.25 (0.13–0.51)	16	8213	0.32 (0.18–0.58)	−0.16	−0.05
20–24	18	9106	0.25 (0.14–0.45)	30	11,325	0.30 (0.19–0.47)	0.17	0.05
25–29	29	7288	0.34 (0.20–0.56)	32	8837	0.35 (0.22–0.56)	−0.03	−0.01
30–39	31	6445	0.61 (0.39–0.96)	44	7648	0.60 (0.40–0.88)	0.02	0.01
40–49	3	574	0.26 (0.27–0.47)	4	690	0.32 (0.11–0.93)	−0.19	−0.06
Region								
North	5	5115	0.07 (0.03–0.18)	21	7236	0.36 (0.22–0.57)	−0.98	−0.35
Northeast	12	4867	0.28 (0.15–0.52)	16	7377	0.24 (0.14–0.41)	0.17	0.04
Southeast	14	7313	0.29 (0.17–0.51)	21	8211	0.37 (0.23–0.58)	−0.22	−0.08
South	46	7694	0.70 (0.51–0.96)	54	8192	0.79 (0.59–1.00)	−0.11	−0.09
Central-West	15	4867	0.63 (0.31–1.30)	14	5697	0.38 (0.17–0.85)	0.66	0.25
Educational level								
Incomplete primary school	6	1574	0.72 (0.29–1.80)	12	2341	0.63 (0.30–1.30)	0.14	0.09
Incomplete secondary school	42	8420	0.61 (0.41–0.90)	60	10,909	0.68 (0.49–0.94)	−0.10	−0.07
Incomplete post-secondary school	22	8442	0.22 (0.13–0.38)	30	10,235	0.28 (0.18–0.44)	−0.21	−0.06
≥ Complete post-secondary school	20	10,792	0.21 (0.12–0.38)	23	12,403	0.21 (0.13–0.36)	0.00	0.00
Race/colour								
White	36	11,078	0.30 (0.20–0.45)	44	12,371	0.33 (0.23–0.48)	−0.09	−0.03
Black	19	2993	0.83 (0.48–1.40)	27	3851	0.80 (0.49–1.3)	0.04	0.03
Asian	2	456	0.96 (0.24–3.8)	3	560	0.94 (0.28–3.10)	0.02	0.02
Mixed (Pardo)	30	14,132	0.24 (0.15–0.38)	48	18,304	0.29 (0.20–0.42)	−0.17	−0.05
Indigenous	1	270	0.48 (0.07–3.30)	1	445	0.34 (0.05–2.40)	−0.41	−0.14

The PPA of the ANC SS test versus the PMTCT HIV test was 84.1% (95% CI: 74.8–91.0) (Table 2). At the regional level, the PPA ranged from 42.9% (95% CI: 9.9–81.6%) in the north to 100.0% (95% CI: 75.0–100.0%) in the central-west (Table 2). The NPA for the ANC SS test versus the PMTCT HIV test was 99.9% (95% CI: 99.9–100.0%), with consistent results across regions. Individual level discrepant results were observed between the PMTCT rapid HIV test results and the ANC SS HIV test results (Table 2). Of the 29,768 negative ANC SS results, 18 (0.09%) were recorded as positive on routine HIV PMTCT testing. By contrast, of the 88 positive ANC SS results, 14 (15.9%) were recorded as negative based on routine HIV PMTCT testing.

**Table 2:** Availability of routine PMTCT HIV status for specimens with ANC surveillance results, Brazil 2010–2012

						Comparison between routine PMTCT (R) and ANC surveillance (S)					
	n	Total	PMTCT HIV test not performed	n (%) missing PMTCT HIV status	Total with routine PMTCT and ANC SS test	R-S+	R+S+	R+S-	R-S-	Positive percent agreement (95% CI)	Negative percent agreement (95% CI)
Overall	36,713	126	5428	732 (2.4%)	29,856	14	74	18	29,750	84.1 (74.8–91.0)	99.9 (99.9–100.0)
Region											
North	7236	21	1900	121 (1.9%)	5115	4	3	2	5106	42.9 (9.9–81.6)	100.0 (99.9–100.0)
Northeast	7377	16	2055	124 (0.02%)	4867	3	5	7	4852	62.5 (24.5–91.5)	99.9 (99.7–99.9)
Southeast	8211	21	681	136 (1.8%)	7313	3	11	3	7296	78.6 (49.2–95.3)	100.0 (99.9–100)
South	8192	54	319	101 (1.3%)	7694	4	42	4	7644	91.3 (79.2–97.6)	99.9 (99.9–100)
Central-West	5697	14	473	250 (4.9%)	4867	0	13	2	4852	100.0 (75.0–100.0)	100.0 (99.9–100.0)

### Magnitude of selection bias in routine HIV PMTCT testing

In total, 86.9% of women accepted HIV testing for PMTCT. By region, the proportion was 74.8% in the north, 73.8% in the northeast, 95% in the southeast, 97.0% in the south, and 90.9% in the central-west.

The HIV prevalence based on ANC SS among those who refused PMTCT testing (*n*=5428) was 0.59% (95% CI: 0.38–0.91%), compared to 0.33% (95% CI: 0.25–0.44%) among non-refusers (Table 3). Among those women missing PMTCT results (*n*=732), the prevalence was 0.48% (95% CI: 0.07–3.3%). The ratio of HIV prevalence among refusers compared to non-refusers was 0.56, whereas the ratio of HIV prevalence among women missing routine PMTCT results compared to those not missing results was 1.26. The percent bias introduced by excluding those who refused routine PMTCT testing from the sample was −10.80%, whereas the percent bias introduced by excluding those who had missing routine PMTCT test results from the sample was 26.3% (Table 3).

**Table 3:** Measure of the effect of PMTCT HIV test status on estimated HIV prevalence, Brazil 2010–2012

		ANC SS test results by availability of PMTCT HIV test			
	Total			Percent bias	Prevalence ratio difference
		PMTCT received	PMTCT not received		
HIV +	119	89	30		
Total	36,016	30,588	5428		
HIV prevalence	0.37 (0.29–0.46)	0.33 (0.25–0.44)	0.59 (0.38–0.91)	−10.80%	0.56
		PMTCT missing	PMTCT not missing		
HIV +	126	1	125		
Total	36,713	732	35,981		
HIV prevalence	0.38 (0.31–0.48)	0.48 (0.07–3.3)	0.38 (0.30–0.48)	26.3%	1.26

### Availability of PMTCT data

The completeness of the PMTCT data was high. The age, date of visit, educational level, and residence had no missing values. The syphilis test results were available in 98.0% of the PMTC records; by region, these results were available in 96.0% of records in the southeast; 98.0% in the north, northeast, and central-west; and 99.0% in the south. The PMTCT HIV test results were nearly 100% complete (97.6%) and ranged from 95.1% in the central-west region to 100% in the northeast. However, there were no data available about previously known HIV-positive status.

## Discussion

This study highlights the potential of the estimates generated by the PMTCT programme for monitoring trends in HIV/AIDS prevalence and for guiding prevention and control interventions in Brazil. When PMTCT HIV testing was the routine standard of care, consistency was found in the HIV prevalence estimates from the PMTCT programme and the ANC SS testing results. However, we observed large differences at the regional level, with the north and central-west presenting greater disparities in overall HIV estimates between the two methods.

Despite a high PNA of 99.9% between individual-level test results from routine testing and ANC SS testing, the overall PPA of 84.1% suggests that additional support quality assurance of HIV testing is required before Brazil can replace the use of the ANC SS with PMTCT programme data. The expected benchmark for transitioning in countries with HIV prevalence among pregnant women of 0.5% is a PPA of 94.7% and a PNA of 99.9% [15]. By region, the PPA was particularly low in the north, a region characterized by a scarcity of technical staff and by the isolation of areas in the interior of the Amazon, which causes difficulties in the provision of quality care to people living with HIV/AIDS. Other studies also reported substantial disagreement between the two testing algorithms, with PPA at 88.5% in Mozambique [16] and 75.9% in Kenya [17]. Of concern, our PPA results suggest that approximately 16 of every 100 HIV positive women receive an HIV negative result. Only 38.2% of women have a second HIV test during pregnancy, which does not ensure that an individual receives the correct result or has access to care [2]. While Brazil has shifted towards the expansion of HIV rapid testing as part of the ANC services, several studies have emphasized issues with the HIV rapid test performance. A study evaluating the ability to perform HIV testing of community healthcare workers (HCWs) in the Amazonas revealed that workers interpreted only 55.8% of HIV and syphilis reference samples correctly [18]. The national external quality assessment programme was rolled out in 2011 to ensure the quality of HIV rapid testing using the dried tube specimens (DTS) method programme [19]. Although this study was conducted in the Amazonas state and may not be representative of the rest of the country, these results highlight the need for continual evaluation and retraining to improve the correct interpretation of test results.

We report an uptake of the PMTCT HIV test of 86.9%, which was slightly lower than desirable for considering PMTCT high testing uptake (90%). This may be partly explained by the ability to optout of HIV testing in the PMTCT programme, which was implemented in 2002 [20]. Nevertheless, we noted regional variations, particularly in the north and northeast of the country, where HIV testing uptake was too low to consider PMTCT data representative of all ANC attendees. Overall selection bias in the PMTCT programme data was borderline (−10.80%) with the threshold established by WHO (from 10 to −10%), i.e., HIV estimates would have decreased by 10.8 percentage points if the results from those who refused HIV testing during PMTCT were removed. This finding indicates that some bias in the prevalence of HIV exists in this survey due to non-acceptance of HIV testing among pregnant women. Overall, there were only 732 women who were missing rapid test results who did not have a documented refusal of the HIV PMTCT test. Interestingly, the HIV prevalence in these women was higher than in those who were not missing results. It is possible that this group included women with known status. The exclusion of these women from the system would increase the HIV estimates by 26.3 percentage points.

The availability of PMTCT data in ANC registers was high with over 90% of the reports with syphilis and HIV testing results. However, reported HIV status at clinic entry, which is a key variable, was not available. In addition, the availability of some variables, such as gravidity, parity, and occupation, were not collected and could not be evaluated. Previous evaluations of the quality of PMTCT data that are routinelly collected presented inconsistent results. In some studies the level of completion of routine data at ANC sites was high [21]. However, other studies underscored data quality limitations in ANC surveillance sites, including the lack of key variables, such as HIV status at enrollment in PMTCT registers [17, 22–24].

This study has specific limitations. PMTC HIV testing is conducted during pregnancy as part of the ANC services and thus at an earlier stage than ANC SS HIV testing, which is conducted in parturient women during maternity service, i.e., labour and delivery. This difference in when the tests are conducted can affect comparisons. Accordingly, for comparison purposes, we selected the first HIV test performed during pregnancy because its uptake was higher (86.9%) than the uptake of the second pregnancy HIV test (38.2%). We did not evaluate health facility testing, and we had insufficient information on the stock of rapid HIV test kits and quality assurance at sites. An evaluation conducted in the state of Ceará showed that 62.5% of prenatal care services at the primary health care level had rapid HIV testing available, and only 37.5% were performing it [25], suggesting the need to strengthen supply chain management and highlighting that rapid HIV testing is not integrated into routine practice. Health service characteristics and management can also affect whether a woman receives a rapid HIV test [26]. We did not assess record keeping practices, which are relevant to understanding how uniform the registers and reporting formats are and to emphasize measures to improve its standardization. In Brazil, prenatal cards are standardized although they vary across regions, which brings heterogeneity to data collected for PMTCT.

## Conclusions

In conclusion, there were consistent results between HIV prevalence from PMTCT data and ANC SS. Despite high NPAs between individual level test results from routine testing and ANC SS for the same patient, the overall PPA was lower than the WHO benchmark for a high level of agreement between the two tests. Testing uptake was slightly lower than the WHO requirement, incurring some selection bias in test acceptance. We also noted the absence of key variables, such as HIV status at booking. Taking these results into account, Brazil must continue to reinforce the quality of PMTCT data collection and of onsite HIV testing in preparation for replacing ANC SS data with PMTCT data for surveillance purposes. Since the last ANC SS was conducted, the Stork Network Initiative has strengthened ANC and expanded the distribution of HIV and syphilis rapid testing among the ANC services [13], which can affect the quality of PMTCT data.

Future comparisons between PMTCT and ANC SS results are recommended before transitioning to an HIV surveillance system based solely on programmatic data. Nevertheless, some regions with better PMTCT data results could be prioritized and pilot the use of PMTCT data for surveillance. Brazil will incorporate HIV testing in the National Survey of Knowledge, Attitudes, and Practices (PCAP), a probability sample survey that collects detailed information on sexual health, HIV status, and other sexually transmitted infections (STIs) among the Brazilian population. This will complement the availability of national HIV estimates of the general population. Upcoming PMTCT data assessments must be more comprehensive and include issues related to HIV testing quality assessment to ensure that the supply of test kits and logistics do not bias results if test kits are not available. In addition, it will be relevant to assess the quality of PMTCT data and to better understand the record keeping practices necessary to improve the standardization of PMTCT reports and optimize monitoring and evaluation. In fact, PMTC-based surveillance data also benefits from training, supervision, and standardization of procedures, an endeavour previously performed for ANCC SS. Finally, the inclusion of a cost-effectiveness component may provide relevant data on savings for the national HIV programme when ANC SS can be substituted and efforts are directed towards generating quality PMTCT programme reports.

## Abbreviations

AIDS: Acquired immune deficiency syndrome
ANC: Antenatal care
CI: Confidence interval
CONEP: National commission for ethics in research
DBS: Dried blood spot samples
DTS: Dried tube specimens
ELISA: Enzyme-linked immunosorbent assay
EQA: External quality assurance
HIV: Human immunodeficiency virus
HIV/AIDS: Human immunodeficiency virus/Acquired immune deficiency syndrome
PCAP: Knowledge, attitudes, and practices
PMTCT: Prevention of mother-to-child transmission
SS: Serosurveys
STI: Sexually transmitted infections
SUS: Sistema único de saúde
WHO: World health organization
